# Informal caregiving patterns and trajectories of psychological distress in the UK Household Longitudinal Study

**DOI:** 10.1017/S0033291718002222

**Published:** 2018-09-12

**Authors:** Rebecca E. Lacey, Anne McMunn, Elizabeth Webb

**Affiliations:** Research Department of Epidemiology and Public Health, University College London, London WC1E 6BT, UK

**Keywords:** Caregiving, longitudinal, panel study, psychological distress, UK Household Longitudinal Study

## Abstract

**Background:**

Approximately seven million people in the UK are engaged in informal caregiving. Informal caregivers are at risk of poorer mental and physical health. However, less is known about how the relationship between the informal caregiving and psychological distress changes over time. The aim of this study was to investigate longitudinal associations between the informal caregiving and psychological distress amongst UK men and women aged 16+.

**Methods:**

Data were analysed from the UK Household Longitudinal Study (UKHLS, *n* = 9368), a nationally representative study of UK households. Longitudinal linear mixed modelling was used to estimate associations between the longitudinal patterns of informal caregiving (non-caregiver/one episode of 1–2 years/intermittent caregiving/3+ years caregiving) and trajectories of psychological distress across seven waves of UKHLS data.

**Results:**

Informal caregiving was not associated with psychological distress for men. Women engaged in long-term (⩾3 years) or intermittent caregiving had higher levels of psychological distress at the point of initiation, compared with women who were not caregivers throughout the study period (3+ years caregiver: regression coefficient 0.48, 95% confidence interval (CI) 0.07–0.89; intermittent caregiver: regression coefficient 0.47, 95% CI 0.02–0.92). Trajectories of psychological distress changed little over time, suggesting a plateau effect for these caregiving women.

**Conclusions:**

Women engaged in long-term or repeated shorter episodes of informal caregiving reported more symptoms of psychological distress than non-caregiving women. Given the increased risk of reporting psychological distress and the increasing importance of the informal care sector, the risk of poorer mental health of informal caregivers should be a priority for public health.

## Introduction

Informal caregiving is a large and important part of the UKs social care sector; currently one in 10 (approximately 7 million) people are engaged in informal caregiving and this is projected to increase by 3.4 million by 2030 (CarersTrust, 2017). Informal caregiving can be a mentally and physically burdensome responsibility which, on average, has a negative effect on health (Vitaliano *et al*., 2003; Pinquart and Sörensen, 2007). Regarding psychological health more specifically, informal caregiving has been associated with increased psychiatric morbidity (Yee and Schulz, 2000), common mental disorders and suicidal thoughts (Stansfeld *et al*., 2014), depression (Schulz *et al*., 1990; Marks *et al*., 2008) and anxiety (Cooper *et al*., 2007). The health effects of informal caregiving are generally more pronounced in women caregivers than in men caregivers (Yee and Schulz, 2000; Amirkhanyan and Wolf, 2006; Pinquart and Sörensen, 2006), and for those providing >10 hours of care per week (Smith *et al*., 2014). This is because women are more likely to be primary caregivers, be engaged in more intense caregiving and report higher caregiver burden (Pinquart and Sörensen, 2006; Arber and Ginn, 2007).

The association between informal caregiving and poorer psychological health is well recognised, however there has been less longitudinal research exploring patterns of informal caregiving and health over time. Firstly, regarding transitions into caregiving, the ‘adaptation hypothesis’ suggests that the demands of caregiving are greatest upon initiation of the caregiving responsibility (Helson, 1964). Indeed, analyses of the British Household Panel Study (BHPS) showed a worsening of psychological distress which was most pronounced shortly following the initiation of caregiving, particularly for caregivers who were engaged in intense caregiving of >20 hours per week (Hirst, 2005). Another study using Health and Retirement Study (HRS) data found an immediate effect of onset of caregiving for an elderly parent followed by a decline in psychological health, but a 2-year lag between the onset of caregiving and physical health decline (Coe and Van Houtven, 2009). We would therefore anticipate the initiation of a caregiving responsibility of any length to be associated with an increase in psychological distress.

The life course approach assumes that caregivers experience change in their health over time (Pearlin, 2010). For instance, caregiving responsibilities and their associated hardships may gradually accumulate over time, labelled as the ‘unexpected career’ by Aneshensel and colleagues (1995) or ‘wear and tear’ by Townsend *et al*. (1989), resulting in a reduction of the caregiver's resources, health and well-being. Consistent with these hypotheses, previous studies showed that long-term caregiving (typically defined as >2 years) was associated with an increase in depressive symptoms and lower levels of well-being. Barnett (2015) used the HRS to analyse the association between caregiving for a parent and trajectories of physical (self-rated health) and psychological health (Center for Epidemiologic Studies Depression Scale, CES-D), finding that both physical and psychological health worsened over time. The findings of Barnett's HRS work were supported by Bookwala (2009) who showed an increase in depression over time for women caregivers in the US National Survey of Families and Households; however men caregiver's depression trajectories declined over time. Also Rafnsson *et al*. (2017) found that long-term care was associated with a decline in quality of life in the English Longitudinal Study of Ageing. Caregiving stress has also been shown to persistent beyond the end of the caregiving responsibility, particularly when a bereavement has occurred (Aneshensel *et al*., 2004; Lee and Gramotnev, 2007). There have not yet been any longitudinal studies which have examined the relationship between the longitudinal patterns of caregiving and trajectories of psychological health.

There are many reasons why informal caregiving might increase psychological distress. Firstly, informal caregiving places strain on personal finances. Carers Trust estimate that 60% of informal caregivers have used all of their personal savings to cover the cost of care and 23% have re-mortgaged their homes or moved to a smaller home (CarersTrust, 2017). Also Stansfeld and colleagues (2014) found in the English Adult Psychiatric Morbidity Survey 2007 that caregivers were more likely to experience debt. The effect of caregiving on finances, might operate through the impact that informal caregiving has the ability to participate in paid employment. Caregiving can be time-consuming and relatively time-inflexible (Hassink and Van den Berg, 2011). Recent research on the UK's Household Longitudinal Study (UKHLS) showed that women who were providing >10 hours per week of care were 2.6 times (95% confidence interval (CI) 1.5–4.8) more likely to exit part-time or 4.5 times (95% CI 2.5–7.9) more likely to exit full-time paid work, compared with women who were not caregivers (Carr *et al*., 2016). No such associations were observed for men, again suggesting that the ways in which caregiving impacts on men and women differs. Informal caregiving may also affect psychological health through constraints on the time available to access social networks and leisure activities (Pearlin *et al*., 1990; Stansfeld *et al*., 2014). Access to social support is known to foster positive psychological well-being (Cohen and Wills, 1985).

The aim of this study was to assess the longitudinal associations between informal caregiving patterns over time and change in psychological distress in a large, longitudinal study in the UK – UKHLS. Our first hypothesis was that a transition into a caregiving responsibility of any length would be accompanied by increased psychological distress, consistent with the ‘adaptation hypothesis’, whereby the demands of caregiving are greatest at the start of the caregiving responsibility. Secondly, caregivers engaged in intermittent periods of caregiving would report higher levels of psychological distress than those engaged in shorter-term, one-off episodes of caregiving. This is again based on the ‘adaptation hypothesis’ as those undertaking repeated episodes of caregiving are likely to have to adapt to each new episode, where the care recipient and/or the level of care need is likely to vary. Thirdly, those undertaking long-term caregiving would experience the highest level of psychological distress and a worsening of psychological distress over time. This is based on the ‘wear and tear’ hypothesis whereby the burden of informal caregiving accumulates over time. Finally, we hypothesised that the relationship between the caregiving and psychological distress would be stronger for women than men.

## Methods

### Data

This study used data from the UKHLS, also known as Understanding Society. UKHLS was initiated in 2009 and is a panel study of a large, nationally representative sample of 40 000 UK households (Lynn, 2009). UKHLS has a stratified, clustered, equal probability sample design. The sample supersedes and includes the BHPS, initiated in 1991. All adults 16 years of age and older in each household are interviewed annually. This study uses data from waves 1–7. Retention of the sample is good; 50 138 individuals participated in wave 1 (82% response rate) and 36 559 remained in wave 7 (88% response rate for wave 6, 73% of wave 1 sample) (Boreham *et al*., 2012; Carpenter, 2017).

### Measures

#### Psychological distress

Psychological distress was indicated by the General Health Questionnaire-12 (GHQ-12), a measure of non-specific psychiatric morbidity, which is widely validated and reliable (Hankins, 2008). As part of the adult self-completion questionnaire in waves 1–7, participants were asked to report recent lack of sleep, inability to concentrate, problems in decision making, strain and feeling overwhelmed, amongst other symptoms. Each GHQ-12 item was scored as not at all (0), no more than usual (1), rather more than usual (2) or much more than usual (3). The GHQ-12 was found to perform well in longitudinal samples with no evidence of retest effects (Pevalin, 2000). A total score was derived for each wave (range 0–36) and kept in continuous form for the analyses.

#### Informal caregiving

Informal caregivers were identified as participants who answered ‘yes’ to either of the following questions in each of waves 1–7:

‘Is there anyone living with you who is sick, disabled or elderly whom you look after or give special help to (for example, a sick, disabled or elderly relative/husband/wife/friend etc.)?’

‘Do you provide some regular service or help for any sick, disabled or elderly person not living with you?’

In order to assess the longitudinal relationship between the informal caregiving and psychological distress, a longitudinal typology of informal caregiving was derived. This had four categories: ‘not caregiving’ comprised of participants who reported not caregiving in all six waves; ‘one episode 1–2 years’ comprised of participants who reported informal caregiving either at only one wave or at two successive waves; ‘intermittent caregiver’ comprised of participants who reported more than one episode of caregiving and ‘3+ years caregiver’ comprised of participants who had at least one episode of caregiving for ⩾3 successive years.

Our sample was restricted to those who were not informal caregivers at wave 1 to enable us to observe caregivers at the initiation of a caregiving episode as we were unable to assess the duration of caregiving prior to the UKHLS survey (see ‘Sample selection’ section below). Also, as we wanted to investigate change in psychological distress over time; our analyses required at least three time points of GHQ data following caregiving initiation. Therefore, our three caregiving categories detailed above included those who initiated their first caregiving episode between waves 2 and 5.

#### Covariates

Our covariates were gender, work status (working, not working), marital status (single, married, separated/divorced or widowed), age in years (continuous), the number of own dependent children in the household (0, 1, 2, 3 or 4+ children), highest obtained educational qualification (no qualifications, secondary school leaving level qualification (e.g. General Certificate of Secondary Education (GCSE) or Ordinary-level (O-level)), tertiary level qualification (e.g. Advanced-level (A-level) or Scottish Highers) or higher level qualification (e.g. university degree or professional qualification)) and National Statistics Socio-Economic Classification of current job (management and professional, intermediate, routine or not working).

### Statistical methods

#### Sample selection

**Fig. 1.**
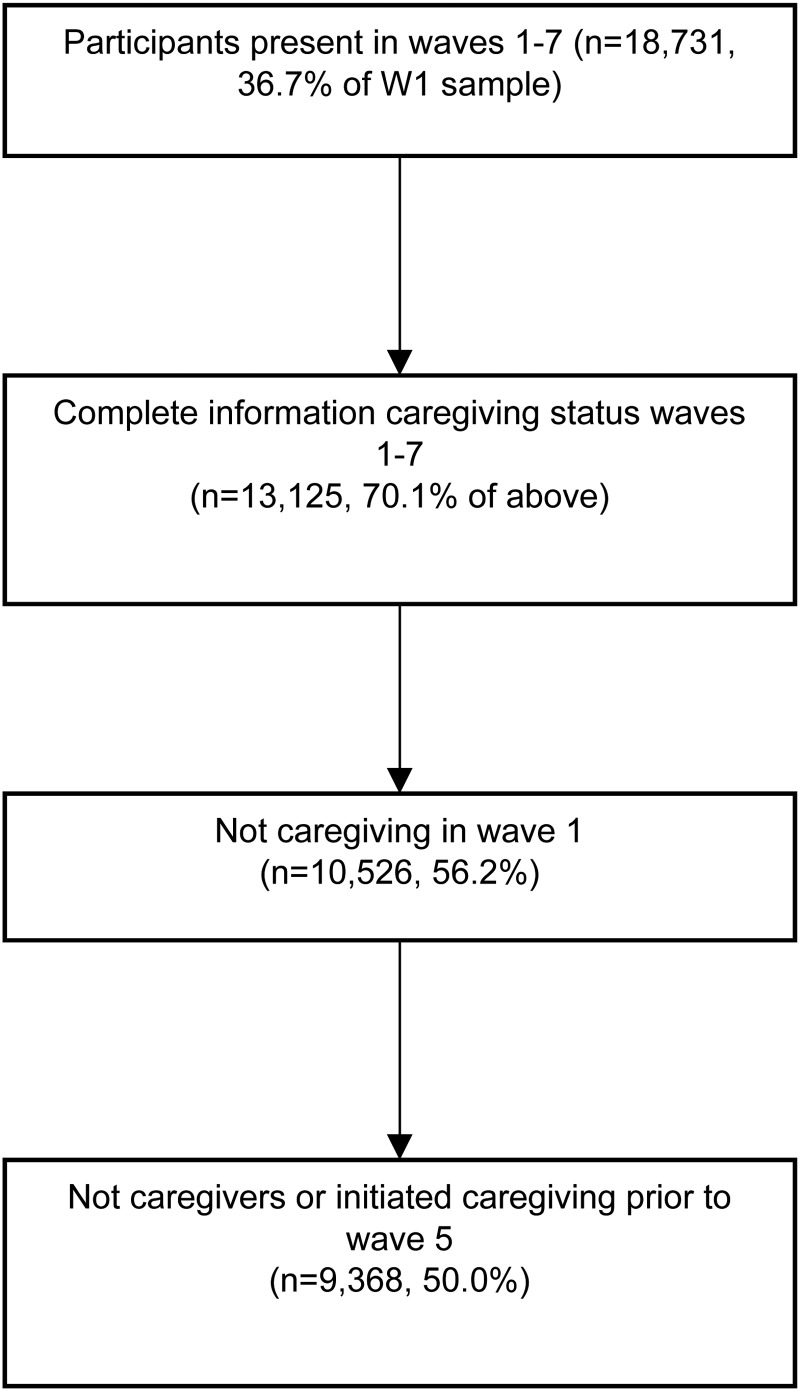
Sample selection procedure.

The analytic sample comprised of participants with complete information on all variables in all waves of UKHLS (*n* = 13 125, see Fig. 1). As described above, to investigate both changes in informal caregiving and psychological distress over time, our analyses were restricted to those who were not informal caregivers at wave 1 (*n* = 10 526), and subsequently to participants who were never caregivers or who initiated their first caregiving episode prior to wave 6 (*n* = 9368) to allow for at least three waves of GHQ-12 data from initiation of caregiving onwards.

#### Longitudinal linear mixed modelling

To model the association between the longitudinal patterns of informal caregiving and change in GHQ, longitudinal linear mixed models were estimated. The intercept for GHQ-12 in these models was set at the point of first observed caregiving initiation for caregivers and was randomly allocated across waves 2–5 for non-caregivers. This approach was used for non-caregivers in order to be consistent with caregivers, for whom the intercept also varied across waves 2–5 depending upon when their first caregiving episode was initiated. We also didn't want to inadvertently bias our findings for non-caregivers by uniformly placing the intercept at a single wave (e.g. wave 2 for all non-caregivers), as this might be affected by study design factors, such as the position of certain questionnaire items, or potential period effects. All covariates were included from the wave prior to the intercept. A series of longitudinal linear mixed models were run. These included: (i) random intercept and fixed slope models without a slope-squared term; (ii) random intercept and fixed slope models with a slope-squared term; (iii) random intercept and random slope models without a slope-squared term; (iv) random intercept and random slope models with a slope-squared term and (v) random intercept and random slope models including an interaction between caregiving and time. All models were estimated separately for men and women as, based on previous literature, women are more likely to be caregivers and to undertake more onerous caregiving responsibilities (Arber and Ginn, 2007), women report more symptoms of psychological distress in general population studies and the relationship between the informal caregiving and health is known to be stronger for women compared with men (Pinquart and Sörensen, 2006). A maximum likelihood algorithm was applied to allow for data from all participants with at least one observed wave of GHQ-12 data to be included in the analyses. 74.9% of our sample had six observed GHQ-12 scores, 15.9% had five, 5.1% had four and 2.6% had three GHQ-12 scores. The high proportion of those with complete GHQ-12 data reflects the fact that, to construct our longitudinal caregiving pattern variable, participants had to have complete information on caregiving, and therefore be present in all waves. Therefore, the figures provided above reflect GHQ-12 item missingness and not wave missingness or attrition. Model fit was assessed by comparing log-likelihood, Akaike Information Criteria and Bayesian Information Criteria values across models, comparing models with the same number of individuals. Only the best fitting nested age-adjusted and covariate-adjusted models are shown. For men, these were age-adjusted and covariate-adjusted random intercept and slope models. For women, these were age-adjusted and covariate-adjusted random intercept and slope models including a slope-squared term. All analyses utilised the survey weights to account for attrition, sampling design and the unequal probability of being sampled. All analyses were conducted using stata version 15 (StataCorp, 2017).

## Results

The characteristics of the analytic sample are presented in Table 1. 24.1% of participants were informal caregivers at some point (9.1% were informal caregiving for one short episode, 6.4% were intermittently caregiving and 8.6% were long-term caregivers for ⩾3 consecutive years). All caregiving patterns were more frequently observed in women, with the greatest gender difference in the ‘3+ years caregiver’ category (10.1% women *v.* 7.0% men). Women reported more symptoms of psychological distress, as indicated by the GHQ-12. Men in the analytic sample were 1.6 years older, on average, than women. There was no gender difference in educational attainment in the analytic sample. Almost a fifth of participants had no educational qualifications but more than a third had higher qualifications. The majority of participants (60.3%) were not living with any of their own dependent children, and this was more common amongst men. Of participants who were living with their own dependent children, most had two or more children (~58% of mothers and fathers). The NS-SEC differed in its prevalence for men and women; men were more likely to be in ‘Management and Professional’ (31.7% men *v.* 24.9% women) or ‘Routine’ occupations (24.2% men *v.* 22.6% women), whereas women were more likely to not be working (36.8% women *v.* 27.4% men). Regarding marital status, men in the analytic sample were more likely to be married and were less likely to be separated, divorced or widowed.

**Table 1.** Characteristics of the study sample

	Men (*n* = 4005) %	Women (*n* = 5363) %	Both (*n* = 9368) %	*p* value gender difference
Caregiving pattern				
Non-caregiver	77.8	74.1	75.9	⩽0.001
1 episode 1–2 years	9.2	9.0	9.1	
Intermittent caregiver	6.0	6.8	6.4	
3+ years caregiver	7.0	10.1	8.6	
GHQ-12, mean	10.0	11.1	10.6	⩽0.001
Age (years), mean	44.5	42.9	43.7	0.002
Educational attainment				
No qualifications	18.4	17.8	18.1	0.454
Secondary	32.8	32.9	32.8	
Tertiary	13.2	11.9	12.5	
Higher qualifications	35.7	37.4	36.6	
Number of dependent children in household				
None	64.5	56.3	60.3	⩽0.001
1	14.9	18.7	16.9	
2	15.1	17.9	16.5	
3	4.3	5.3	4.8	
4+	1.2	1.8	1.5	
NS-SEC				
Management and professional	31.7	24.9	28.2	⩽0.001
Intermediate	16.8	15.7	16.2	
Routine	24.2	22.6	23.3	
Not working	27.4	36.8	32.3	
Work status				
Working	70.5	59.7	64.9	⩽0.001
Not working	29.5	40.3	35.1	
Marital status				
Single, never married	30.0	30.1	30.0	⩽0.001
Married	65.2	58.6	61.8	
Separated/divorced	4.0	8.7	6.4	
Widowed	0.8	2.7	1.8	

Weighted percentages or means shown.

Further description of the characteristics of participants in each caregiving category is shown in Supplement 1. There was little difference in the mean GHQ-12 scores of men by the caregiving group. However, women in the ‘Intermittent caregiver’ and ‘3+ years caregiver’ groups had higher GHQ-12 scores, on average, than women who were not caregivers or who were engaged in shorter-term caregiving. Men and women who were engaged in caregiving were older than men and women who weren't caregiving. On average, men in the ‘Intermittent caregiver’ group were the oldest, however amongst women those in the ‘3+ years caregiver’ group were the oldest, on average. Men and women who had done caregiving of any duration were less likely to have any educational qualifications. Men in the ‘3+ years caregiver’ group were the least likely to have dependent children in the household. However, women who did a single episode of caregiving were the most likely to have no dependent children living with them. Men and women in the ‘Intermittent caregiver’ group were the most likely to have dependent children in the household. Men who were ‘Intermittent’ or longer-term caregivers were the most likely to not be working and non-caregiving men were the most likely to be in ‘Management or professional’ occupations. A similar pattern was observed for women. Finally, regarding marital status, the ‘Intermittent caregiving’ group contained the largest proportion of married or widowed men, whereas the long-term caregiving women were the most likely to be married.

### Association between the caregiving patterns and trajectories of psychological distress

Table 2 shows the results of the random intercept and slope models for UKHLS men. At intercept (point of initiation of first caregiving episode amongst caregivers; randomly assigned amongst non-caregivers), men had a mean GHQ-12 score of 10.75 (95% CI 10.36–11.15), after accounting for age. No differences were observed in the intercept GHQ-12 score by caregiving pattern. Also, there was only a very small observed change in GHQ-12 scores over time in the whole sample (−0.03, 95% CI −0.06 to −0.002) but not by caregiving pattern. There was little change in these findings after additionally adjusting for the number of dependent children, educational attainment, NS-SEC, marital status or work status. Inclusion of a caregiving-time interaction term worsened model fit, indicating that the association between the informal caregiving pattern and GHQ did not change over time.

**Table 2.** Association between the caregiving patterns and GHQ over time for UKHLS men (*n* = 4005)

	Age-adjusted	Covariate-adjusted
Fixed effects		
GHQ-12 intercept^a^	10.75 (10.36, 11.15)	10.39 (9.74, 11.04)
Caregiving pattern		
Non-caregiver	Ref	Ref
1 episode 1–2 years	0.12 (−0.33, 0.56)	0.10 (−0.34, 0.54)
Intermittent caregiver	0.44 (−0.10, 0.97)	0.37 (−0.17, 0.90)
3+ years caregiver	0.16 (−0.32, 0.65)	0.22 (−0.26, 0.71)
Slope^b^	−0.03 (−0.06, −0.002)	−0.03 (−0.06, −0.001)
Random effects		
Variance (slope)	0.26 (0.22, 0.31)	0.26 (0.22, 0.31)
Variance (intercept)	10.51 (9.43, 11.72)	10.32 (9.26, 11.49)
Covariance (slope, intercept)	−0.31 (−0.47, −0.14)	−0.30 (−0.46, −0.14)
Variance (residuals)	9.98 (9.39, 10.62)	9.99 (9.39, 10.62)
Model fit indices		
Log likelihood	−62 006.2	−61 984.4
AIC	124 032.4	124 010.9
BIC	124 114.1	124 182.4

a Intercept set at point of initiation of first caregiving episode or randomly assigned to waves 2–5 for non-caregivers.

b Slope term represents change in GHQ-12 score per wave.

AIC, Akaike Information Criterion; BIC, Bayesian Information Criterion.

For women, the best fitting models were random intercept and slope models including the addition of a time-squared term (Table 3). The GHQ-12 intercept was higher for women (mean GHQ-12 = 12.04, 95% CI 11.64–12.43) than for men. After accounting for age differences, women who were engaged in ‘Intermittent caregiving’ or ‘3+ years caregiving’ had higher GHQ-12 scores upon initiating caregiving (intermittent caregiving: 0.54 higher, 95% CI 0.08–1.00; 3+ years caregiving: 0.46, 95% CI 0.04–0.87). These estimates were largely unchanged following inclusion of other covariates (number of dependent children, educational attainment, NS-SEC, marital and work status), although the intercept coefficient was attenuated (mean GHQ score at intercept: 11.34, 95% CI 10.70–11.98). Also, the slope-squared term representing quadratic change in GHQ-12 was significant for women (−0.01, 95% CI −0.02 to −0.001). Similar to men, the relationship between the informal caregiving pattern and GHQ did not change over time.

**Table 3.** Association between the caregiving patterns and GHQ over time for UKHLS women (*n* = 6113)

	Age-adjusted	Covariate-adjusted
Fixed effects		
GHQ-12 intercept^a^	12.04 (11.64, 12.43)	11.34 (10.70, 11.98)
Caregiving pattern		
Non-caregiver	Ref	Ref
1 episode 1–2 years	0.09 (−0.34, 0.03)	0.07 (−0.35, 0.50)
Intermittent caregiver	0.54 (0.08, 1.00)	0.47 (0.02, 0.92)
3+ years caregiver	0.46 (0.04, 0.87)	0.48 (0.07, 0.89)
Slope^b^	0.002 (−0.03, 0.03)	0.002 (−0.03, 0.03)
Slope^2^	−0.01 (−0.02, −0.001)	−0.01 (−0.02, −0.001)
Random effects		
Variance (slope)	0.23 (0.19, 0.28)	0.23 (0.19, 0.28)
Variance (intercept)	11.78 (10.78, 12.86)	11.40 (10.44, 12.44)
Covariance (slope, intercept)	−0.08 (−0.24, 0.07)	−0.07(−0.23, 0.08)
Variance (residuals)	14.38 (13.69, 15.11)	14.38 (13.69, 15.11)
Model fit indices		
Log likelihood (model)	−69 991.0	−69 942.1
AIC	140 004	139 928.3
BIC	140 097.1	140 114.3

a Intercept set at point of initiation of first caregiving episode or randomly assigned to waves 2–5 for non-caregivers.

b Slope term represents change in GHQ-12 score per wave.

## Discussion

### Summary of findings

Using a large UK longitudinal study, the UKHLS, we found that around a fifth of participants reported becoming an informal caregiver at some point over the 7-year period studied. Consistent with many previous population studies, GHQ-12 scores were higher for women compared with men. Informal caregiving was more common amongst women and women were also more likely to participate in long-term caregiving than men. We observed no differences in psychological distress for men by different caregiving patterns. However, women who were engaged in long-term caregiving (3+ years) or who were intermittent caregivers reported modestly more symptoms of psychological distress than women non-caregivers at the point at which they first initiated informal caregiving. There was little evidence that after initiating caregiving that trajectories of psychological distress were different from non-caregivers over time, therefore suggesting that levels of psychological distress remained raised for these caregiving women with no evidence of increase or decline over time. The associations observed in this study remained after inclusion of sociodemographic characteristics such as age, educational attainment, the number of dependent children, social class, work and partnership status.

### Interpretation of findings

Consistent with much of the literature, women in the UKHLS are more likely than men to be providers of informal care and also to be caregiving over longer periods of time. Previous work has shown that women are more likely to be primary caregivers than men, and also to undertake more onerous caregiving responsibilities, such as caregiving for people with more complex care needs (Pinquart and Sörensen, 2006; Arber and Ginn, 2007). As such, women's caregiving responsibilities are likely to require a longer-term commitment and likely to be accompanied by both a greater level of ‘adaptation’ and long-term ‘wear and tear’. Related to this, women caregivers tend to report poorer health than men caregivers (Pinquart and Sörensen, 2006), and this is consistent with our findings also. In this study, longitudinal patterns of informal caregiving were not associated with psychological distress. This finding was in contrast to Bookwala's (2009) study where, on average, informal caregiving men displayed a decrease in depressive symptoms over time. The author purports that there may be gender differences in the psychological response to caregiving; women may be more likely to experience psychological ‘wear and tear’ over time, but men undertaking caregiving responsibilities over a similar length of time are more likely to adapt. However, this is likely to be confounded by the intensity of the caregiving responsibilities and the larger investment in other time-demanding social roles such as parenting. Bookwala's study differs from ours since it is based upon a small US sample of caregivers providing care to parents, whereas our study is based upon a broader nationally representative sample of UK caregivers aged 16 and over.

Women in this study who were long-term or intermittent caregiving reported more symptoms of psychological distress at the wave in which caregiving was first reported. These associations were small in size. In general, longitudinal studies of informal caregiving and health tend to show smaller effect sizes than cross-sectional studies (Vlachantoni *et al*., 2013). Our findings for long-term and intermittent caregiving are not consistent with the ‘adaptation hypothesis’ (Helson, 1964), as this hypothesis suggests that caregiver's psychological distress returns to pre-caregiving levels in time. In contrast, our findings suggested that psychological distress was higher for long-term and intermittent caregiving women at initiation but then no differences were observed in the slopes suggesting no adaptation in psychological distress over time. Whilst these findings might initially suggest that women in these two informal caregiving groups are more psychologically distressed prior to or at the onset of caregiving, it's also possible that there is a lag between the onset of a caregiving need and self-definition as an informal caregiver. This would particularly be the case for caregivers providing longer-term care needs, for example caring for someone with a degenerative condition. We found no suggestion that trajectories of psychological distress for caregivers changed over time in comparison with non-caregivers for men or women, or were particularly worse for those undertaking the longest periods of caregiving. Our findings in this respect are therefore also not consistent with Townsend's ‘wear and tear’ hypothesis whereby the burden of informal caregiving accumulates over time (Townsend *et al*., 1989). However, our findings are consistent with Lawton *et al*. (2000) who found that caregiving for long periods of time was not associated with a worsening of caregiver well-being. However, our findings are in contrast to a few other studies, including work by Barnett (2015) in which women in the HRS did not experience a decline in psychological health over time in response to caregiving alone, but the combination of caregiving with other social roles, such as partnerships and paid employment contributed significantly to a decline in psychological health over time. More studies are required which consider the combination of informal caregiving with other social roles, such as paid employment, parenthood and partnerships over time. Further research is also required to investigate potential mediators of the association between the intermittent and long-term caregiving on psychological distress for women, perhaps exploring the role of financial strain and social support (Stansfeld *et al*., 2014).

### Strengths and limitations

Whilst we had information on caregiving within and outside the household, this would have been difficult to disentangle over time. Evidence suggests that caregiving for a household member is more stressful and therefore associated with poorer psychological health than caregiving for someone outside of the household (Barrow and Harrison, 2005). Secondly, studies of informal caregiving are frequently confounded by the difference between the need for care and care provision. For instance, Amirkhanyan and Wolf (2006) found that having a parent who required care increased the likelihood of depression, regardless of whether informal care was provided. Similarly Bobinac and colleagues (2010) found that this ‘family effect’ showed an association of a similar magnitude as the ‘caregiving effect’ on well-being. Unfortunately, we were not able to distinguish between the family and caregiving effects using the UKHLS. We were also unable to explore the effects of caregiving intensity in combination with caregiving patterns in this study as this would have required the derivation of an extremely complex typology of longitudinal caregiving patterns, resulting in small cell sizes and consequently low statistical power. A further limitation of our study was the use of complete case analyses. Our analyses included UKHLS participants with complete caregiving data across all seven waves and complete covariate data from the wave at which caregiving was first reported (randomly allocated across waves 2–5 for non-caregivers). As such, our findings are likely to be underestimated as participants who remain in longitudinal studies tend to be healthier and more socially advantaged than those with missing information (Abraham and Russell, 2004). However, we minimised missing information on GHQ-12 by applying maximum likelihood estimation in the longitudinal linear mixed modelling. This method uses all available information to model change in GHQ-12 over time. Further, the GHQ-12 is not a measure of specific psychiatric diagnoses but a screening tool for non-specific psychiatric morbidity; however our findings are consistent with studies utilising clinically-validated measures. Finally, it is possible that participants had been informal caregivers prior to the initiation of the UKHLS, and this is a potential limitation of any study which uses a general purpose population sample to explore first caregiving transitions. To mitigate this limitation we restricted our analyses to caregivers who were observed as not providing informal care for at least one wave prior to the point we defined as initiation of caregiving.

Despite these limitations, our study also has a number of strengths. These include the use of a large, longitudinal dataset – the UKHLS and therefore the ability to investigate longitudinal patterns in informal caregiving, the first study of this kind. Also, the availability of repeated measures of GHQ-12 enabled the investigation of trajectories in psychological distress over time. Finally, our sample of caregivers were aged 16+ and therefore were not restricted to middle-aged or older caregivers or caregivers to people with specific health conditions, for example Alzheimer's disease, as in many previous studies.

## Conclusions

In summary, using a large longitudinal study of UK men and women we found that women engaged in long-term (3+ years) or intermittent caregiving reported slightly higher levels of psychological distress when caregiving was first reported compared with women who were non-caregivers. Psychological distress did not increase over time for informal caregiving men or women. Given the initially increased risk of psychological distress of informal caregivers, particularly women and those undertaking longer-term caregiving responsibilities, the potentially poorer mental health of informal caregivers merits public health promotion efforts.

## Supplementary material

For supplementary material accompanying this paper visit https://doi.org/10.1017/S0033291718002222. click here to view supplementary material
