# Supplementary material for: Informal caregiving patterns and trajectories of psychological distress in the UK Household Longitudinal Study
Source: Psychol Med. 2018 Sep 12;49(10):1652–60. doi: 10.1017/S0033291718002222 (PMC6601356; doi:10.1017/S0033291718002222)
Supplement: Supplementary file 1 [file S0033291718002222sup001.docx]

Supplement 1 GHQ-12 and covariates by caregiving pattern for UKHLS men and women

|  | **Men** | | | | **Women** | | | |
| --- | --- | --- | --- | --- | --- | --- | --- | --- |
|  | **Non caregiver**  **%** | **1 episode**  **1-2 years**  **%** | **Intermittent caregiver**  **%** | **3+years caregiver**  **%** | **Non caregiver**  **%** | **1 episode**  **1-2 years**  **%** | **Intermittent caregiver**  **%** | **3+years caregiver**  **%** |
| GHQ-12, mean | 10.0 | 10.3 | 10.6 | 10.3 | 11.1 | 11.1 | 11.5 | 11.5 |
| Age (years), mean | 43.2 | 47.8 | 50.8 | 49.5 | 41.2 | 47.3 | 48.0 | 48.2 |
| Educational attainment |  |  |  |  |  |  |  |  |
| No qualifications | 16.9 | 18.9 | 32.7 | 21.8 | 17.0 | 20.0 | 22.3 | 19.1 |
| Secondary | 32.3 | 37.0 | 32.2 | 32.7 | 31.2 | 37.7 | 36.9 | 38.7 |
| Tertiary | 13.3 | 16.2 | 9.6 | 12.1 | 12.9 | 9.0 | 8.6 | 9.1 |
| Higher qualifications | 37.6 | 27.9 | 25.6 | 33.5 | 39.0 | 33.4 | 32.1 | 33.2 |
| Number of dependent children in household |  |  |  |  |  |  |  |  |
| None | 63.0 | 70.1 | 61.3 | 76.8 | 55.5 | 61.1 | 58.4 | 57.1 |
| 1 | 15.8 | 10.2 | 13.5 | 13.0 | 19.7 | 13.0 | 16.8 | 17.7 |
| 2 | 15.5 | 15.6 | 19.1 | 7.0 | 18.4 | 19.4 | 12.7 | 16.0 |
| 3 | 4.5 | 3.5 | 3.9 | 2.6 | 5.2 | 4.4 | 6.6 | 6.2 |
| 4+ | 1.2 | 0.6 | 2.3 | 0.6 | 1.3 | 2.2 | 5.5 | 3.0 |
| NS-SEC |  |  |  |  |  |  |  |  |
| Management & professional | 32.8 | 28.0 | 23.9 | 31.1 | 26.1 | 21.1 | 19.5 | 23.6 |
| Intermediate | 17.4 | 15.8 | 14.9 | 12.4 | 15.5 | 17.1 | 15.2 | 16.9 |
| Routine | 25.2 | 24.1 | 19.0 | 17.2 | 23.5 | 20.1 | 21.7 | 18.9 |
| Not working | 24.6 | 32.1 | 42.3 | 39.4 | 35.0 | 41.7 | 43.6 | 40.6 |
| Work status |  |  |  |  |  |  |  |  |
| Working | 73.3 | 63.8 | 56.5 | 59.6 | 61.1 | 55.7 | 53.7 | 56.9 |
| Not working | 26.7 | 36.2 | 43.5 | 40.4 | 38.9 | 44.3 | 46.3 | 43.1 |
| Marital status |  |  |  |  |  |  |  |  |
| Single, never married | 31.6 | 24.3 | 22.8 | 24.9 | 33.4 | 20.3 | 23.1 | 19.2 |
| Married | 64.1 | 67.1 | 71.7 | 69.4 | 55.7 | 66.3 | 61.6 | 71.0 |
| Separated/divorced | 3.6 | 7.6 | 1.9 | 5.3 | 8.2 | 11.0 | 10.5 | 8.8 |
| Widowed | 0.6 | 1.0 | 3.6 | 0.4 | 2.7 | 2.5 | 4.8 | 1.1 |

Weighted percentages or means shown
